# Investigation of a Novel Hydrogen Depressurization Structure Constituted by an Orifice Plate with Tesla-Type Channels

**DOI:** 10.3390/ma15144918

**Published:** 2022-07-14

**Authors:** Bei Li, Yu Liu, Jiaqing Li, Bin Liu, Xingxing Wang, Guanyu Deng

**Affiliations:** 1 School of Mechanical Engineering, Nantong University, Nantong 226019, China; 2010110241d@stmail.ntu.edu.cn (B.L.); wangxx@ntu.edu.cn (X.W.); 2 School of Mechanical, Materials, Mechatronic and Biomedical Engineering, University of Wollongong, Wollongong, NSW 2522, Australia; 3 College of Chemical Engineering, Fuzhou University, Fuzhou 350116, China; 4 School of Mechanical Engineering, Shijiazhuang Tiedao University, Shijiazhuang 050043, China; liubin@stdu.edu.cn

**Keywords:** hydrogen, hydrogen fuel cell, depressurization, orifice plate structure, computational fluid dynamics, numerical model

## Abstract

A hydrogen depressurization system is required to supply the hydrogen to the fuel cell stack from the storage. In this study, a Tesla-type depressurization construction is proposed. Parallel Tesla-type channels are integrated with the traditional orifice plate structure. A computational fluid dynamics (CFD) model is applied to simulate high-pressure hydrogen flow through the proposed structure, using a commercial software package, ANSYS-Fluent (version 19.2, ANSYS, Inc. Southpointe, Canonsburg, PA, USA). The Peng–Robinson (PR) equation of state (EoS) is incorporated into the CFD model to provide an accurate thermophysical property estimation. The construction is optimized by the parametric analysis. The results show that the pressure reduction performance is improved greatly without a significant increase in size. The flow impeding effect of the Tesla-type orifice structure is primarily responsible for the pressure reduction improvement. To enhance the flow impeding effect, modifications are introduced to the Tesla-type channel and the pressure reduction performance has been further improved. Compared to a standard orifice plate, the Tesla-type orifice structure can improve the pressure reduction by 237%. Under low inlet mass flow rates, introduction of a secondary Tesla-type orifice construction can achieve better performance of pressure reduction. Additionally, increasing parallel Tesla-type channels can effectively reduce the maximum Mach number. To further improve the pressure reduction performance, a second set of Tesla-type channels can be introduced to form a two-stage Tesla-type orifice structure. The study provides a feasible structure design to achieve high-efficiency hydrogen depressurization in hydrogen fuel cell vehicles (HFCVs).

## 1. Introduction

Contemporarily, the transportation sector represents more than one-quarter of carbon gas emissions [1]. An increasing application of hydrogen is considered a potential strategy to gradually fulfill net-zero carbon emissions in the transportation sector [2]. Hydrogen is also considered the ultimate conventional energy source of the 21st century due to its cleanness and sustainability [3]. Therefore, the application of hydrogen fuel cell vehicles (HFCVs) has attracted significant interest [4]. A practical and economical method for HFCV applications is by using pressurized hydrogen storage tanks, as the volumetric energy density of gaseous hydrogen is extremely low [5]. To improve the travelling distance of HFCVs, the tank pressure has been continuously increased during the development of HFCVs. However, the optimal working pressure of the fuel cell tends to be low, leading to the growing demand on the performance of depressurization system [6,7,8].

In recent years, a number of studies on high-pressure gas depressurization have been undertaken. Luo and his colleagues [9] developed a pressure reducing valve that has a fixed pressure ratio. The pressure and leakage characteristics were theoretically analyzed through simulations. The results show that as the operating pressure increases, the pressure ratio reduces to the designed value. Ulanicki et al. [10] investigated the oscillation of pressure reducing valves (PRVs) at low flow rates. The study was motivated by an industrial case analysis. The purpose of this study is eliminating pressure fluctuation. The results show that the PRV is less stable for small valve openings. Binod and his group [11] utilized a computational fluid dynamic (CFD) model to investigate the transient process in pressure regulation and shut-off valves. Okhotnikov et al. [12] studied pressure drops and steady flow torques of the valve at various flow rates and orifice openings by the CFD method, and relative information, such as the discharge co-efficient and flow jet angles dependencies on the orifice opening, was obtained from this study. Jin and his group [13,14] designed a high-level multi-stage PRV (HMPRV) for hydrogen depressurization in hydrogen refueling stations. It was found that the HMPRV can successfully control the gas pressure and working temperature and is less prone to block flow. In their previous work, the mechanisms of pressure reduction and energy conversion was investigated based on a novel PRV with an orifice plate. In order to optimize valve performance, a parametric study on the throttling portion of a HMPRV was undertaken by Hou and his team [15]. It was found that larger hydrogen kinetic energy causes a stronger turbulent vortex, higher energy consumption, larger multistage injection casing diameter, injection-plate diameter, and pressure ratio. Chen et al. [16] investigated the effects of valve openings on flow characteristics in detail. It was found that larger pressure and velocity gradients mainly appeared at the throttling components for all valve openings. A larger valve opening resulted in more energy consumption. Chen et al. [17,18] simulated the compressible turbulent flow in an HMPRV using CFD software ANSYS-Fluent to analyze the noise and energy consumption. Liu and his group [19] studied the hydrogen flow through a perforated plate in a pressure-reducing system based on a CFD model. The thermodynamic properties of hydrogen were described using a real fluid equation of state (EoS). In addition, the effect of the types of perforated plate was investigated. The results show that the size of the perforated plate has a significant effect on the hydrogen flow.

The above-mentioned PRVs comprise rotating parts with complex structures which will cause excessive turbulence and noise; the complexity of the structures will also result in manufacturing difficulties. In recent years, the Tesla valve [20] has attracted growing attention in relation to pressure depressurization, as it can cause a significant pressure drop when the flow of fluids is reversed. Tesla valves have a fixed geometry with no moving parts, therefore, they may have a longer lifetime and can facilitate mass production. A large number of investigations have been undertaken on using Tesla valve for pressure reduction, mainly focusing on structure optimization. The Tesla valve shape is optimized through two-dimensional (2D) CFD simulations combined with an optimization procedure [21]. A three-dimensional (3D) parametric model is proposed for the Tesla valve by Zhang et al., and his group optimized the geometric relationships of Tesla valve [22]. De Vries et al. [23] designed a new Tesla valve and symmetrically integrated it into a single rotating pulsating heat pipe (PHP). They then investigated the flow characteristics and thermal performance of the PHP. Bao et al. [24] designed a novel Tesla valve with a special tapering/widening structure, analyzed and compared it with other types of Tesla valve, to find which showed a superior absolute pressure drop ratio. Monika et al. [25] developed a multi-stage Tesla valve configuration to enhance heat transfer. Zhang and his colleagues [26] designed a multistage pressure-reducing valve; the valve combined a Tesla-type orifice valve and a sleeve pressure structure valve. In this study, the influences of working parameters on fluid pressure and velocity distributions were analyzed. Qian et al. [27,28] performed simulations for hydrogen reverse flow in a multi-stage Tesla valve. They summarized the power–law relationship in the flow rate, the number of stages, and the pressure ratio, and evaluated them using Mach number, turbulent dissipation rate, and blown-barrel loss as criterions. Jin and his team [29] studied the influence of different structural parameters of a single-stage Tesla valve on the hydrogen pressure reduction. The results show that a smaller hydraulic diameter, a smaller inner curve radius, and a larger valve angle could provide a higher pressure drop at a larger inlet velocity. Qian and Jin et al. [27,29] predicted the physical properties of the ideal gas EoS during simulation. However, since the ideal gas EoS does not take into account the effect of intermolecular potential energy, it will produce large errors under high-pressure conditions.

As mentioned above, several studies were conducted on Tesla valves as well as multi-stage pressure-reducing structures. However, studies connecting the Tesla valve to the traditional perforated plate structure are seldom found. The actual gas EoS refers to the mathematical expression of the functional relationship between the state parameters when a certain amount of gas reaches equilibrium state. The ideal gas completely ignores the interaction between gas molecules and cannot explain phenomena such as gas–liquid change and throttling in which molecular forces play an important role. However, the hydrogen depressurization system operates at very high pressures under which the ideal gas EoS may produce large errors. Additionally, Peng–Robinson (PR) EoS, a real gas EoS, is simple to calculate and accurate to the physical property of pure gas. Some studies on flow and heat transfer under complex conditions offer a great help to this paper’s investigation of the flow through the Tesla-type orifice structure. Rezaei et al. [30] studied electro-osmotic flow of an aqueous solution of NaCl using the molecular dynamics simulation to investigate the effects of the electric field and temperature on the flow properties. Toghraie et al. [31] conducted a simulation to study boiling heat transfer through a volume fraction (VOF) method, and they also studied the quench phenomena through a fluid jet on a hot horizontal surface. Li and his co-workers [32] investigated the fluid flow and heat transfer using two-phase approach mixed convection of a non-Newtonian nanofluid in a porous H-shaped cavity. These studies showed that the simulation technology was able to simulate the fluid flow through complex geometric conditions.

In this paper, a 3D CFD model integrated with the PR real gas EoS was proposed to investigate the pressure decrease in a new Tesla-type orifice structure. The traditional perforated plate structure is displaced by a flow channel with Tesla valves in the hydrogen pressure reduction system. The structure (Tesla-type orifice plate structure) consists of multiple Tesla valves in parallel to achieve higher pressure reduction. In addition, the optimization for the Tesla valve is undertaken to improve the characteristics of the backflow impact in its flow channel. Furthermore, a two-stage Tesla-type orifice plate structure, which comprises two Tesla in series, is introduced. The effects of structural parameters on the flow characteristics are investigated to obtain better depressurization performance. This research offers technical support for HFCVs.

## 2. Numerical Methods

### 2.1. Governing Equations

The CFD software ANSYS-Fluent is employed for the numerical solution. ANSYS-Fluent uses the finite volume method to discretize the governing differential equations of fluid flow based on the Navier–Stokes (N–S) equation, which involves the solution of mass, momentum, and energy conservation equations [33], as expressed in Equations (1)–(3).

(1) $$\frac{\partial \rho }{\partial t}+\nabla \cdot (\rho \nu )=0$$

(2) $$\frac{\partial }{\partial t}(\rho \nu )+\nabla \cdot (\rho \nu )=-\nabla \cdot p+\nabla \cdot \tau +\rho g$$

(3) $$\frac{\partial }{\partial t}(\rho E)+\nabla \cdot [\nu (\rho E+p)]=\nabla \cdot (k_{eff}\Delta T-\tau \nu )$$

where *ρ* is the density, *t* the time, ***ν*** the velocity vector, ***p*** the pressure vector, *τ* the viscous stress tensor, ***g*** the gravitational acceleration, *E* the total energy per unit control body, and *k_eff_* the effective thermal conductivity.

### 2.2. Turbulence Model

An appropriate turbulence model is crucial to simulate hydrogen flow with high compressible pressure gradient. As the influence of the compressibility on turbulence dissipation cannot be explained by the standard *k-ε* model [34], the realizable *k-ε* model is applied in this work. In practice, the realizable *k-ε* turbulence model [35] has been successfully used in various flows, such as separated flows, channel and boundary layer flows, and rotating homogeneous shear flows. Particularly, the realizable *k-ε* model can better predict the diffusion rates of axisymmetric and planar jets. The realizable *k-ε* model is described as:

(4) $$\frac{\partial }{\partial t}\left(\rho k\right)+\frac{\partial }{\partial x_{i}}\left(\rho ku_{i}\right)=\frac{\partial }{\partial x_{j}}\left[\left(\mu +\frac{\mu_{t}}{\sigma_{k}}\right)\frac{\partial k}{\partial x_{j}}\right]+G_{k}+G_{b}-\rho \varepsilon -Y_{M}+S_{k}$$

(5) $$\frac{\partial }{\partial t}\left(\rho \varepsilon \right)+\frac{\partial }{\partial x_{j}}\left(\rho \varepsilon u_{j}\right)=\frac{\partial }{\partial x_{j}}\left[\left(\mu +\frac{\mu_{t}}{\sigma_{\varepsilon }}\right)\frac{\partial \varepsilon }{\partial x_{j}}\right]+\rho C_{1}S\varepsilon -\rho C_{2}\frac{\varepsilon^{2}}{k+\sqrt{\upsilon \varepsilon }}+C_{1\varepsilon }\frac{\varepsilon }{k}C_{3\varepsilon }G_{b}+S_{\varepsilon }$$

where *G_k_* denotes the generation of turbulence kinetic energy owing to the average velocity gradients; *G_b_* represents the generation of turbulence kinetic energy owing to buoyancy; *Y_M_* is the contribution of the fluctuating dilatation incompressible turbulence to the overall dissipation rate; *C*_2_ and *C*_1ε_ are constants; *σ_k_* and *σ_ε_* are the turbulent Prandtl numbers for *k* and *ε*, respectively; and *S_K_* and *S_ε_* are user-defined source terms.

### 2.3. PR EoS

An accurate prediction of the thermodynamic properties of the fluid is essential to achieve satisfactory accuracy in the CFD model. In this study, a real gas Eos, i.e., the PR EoS, is applied to predict better thermodynamic properties of high-pressure hydrogen. The PR EoS [36] is illustrated by:

(6) $$P=\frac{RT}{\nu -b}-\frac{a(T)}{\nu^{2}+2b\nu -b^{2}}$$

(7) $$b=0.0778\frac{RT_{c}}{P_{c}}$$

(8) $$a(T)=0.45724\frac{R^{2}T_{c}^{2}}{P_{c}}[1+n{\left(1-{\left(T/T_{c}\right)}^{0.5}\right]}^{2}$$

(9) $$n=0.37464+1.54226\omega -0.26993\omega^{2}$$

where *R* represents the universal gas constant, *ν* the molar volume, *P_c_* the critical pressure, *T_c_* the critical temperature, and *ω* the eccentricity factor of the gas.

**Figure 1:**
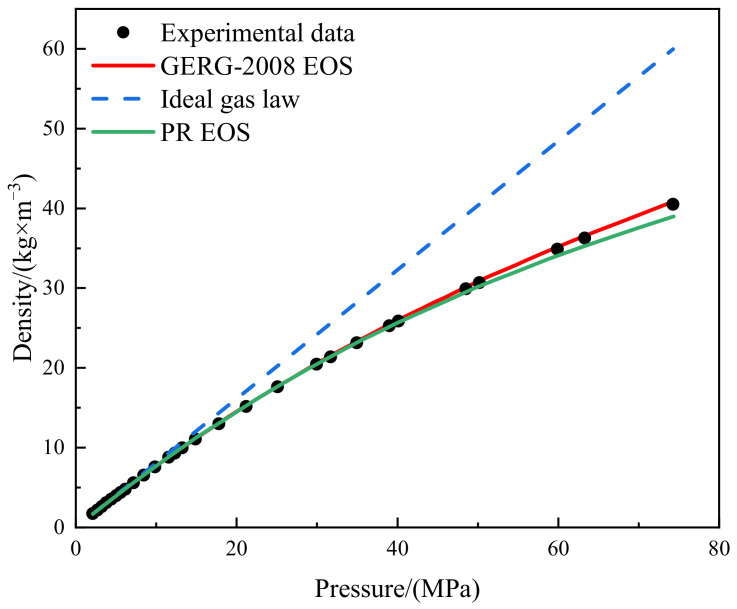
Comparison between different equations of state and experimental hydrogen thermophysical parameters.

Hydrogen densities at various conditions, which are adopted from the experimental data of Michels et al. [37] are utilized to evaluate the accuracy of the PR EOS. Figure 1 shows the comparison between experimental data and EoS predictions. It is obvious that the ideal gas EoS will cause large discrepancies at high pressures. The densities calculated by PR EOS are consistent with the measurements and the maximum relative error is 3.8%. GERG-2008 (Groupe Européen de Recherches Gazières) EoS [38] performs slightly better than the PR EoS at higher pressures. However, comparing to the PR EoS, it is much more time-consuming to solve the GERG-2008 EoS at runtime during the 3D CFD simulations; therefore, it is an adequate choice to employ PR EoS in the simulations to ensure acceptable accuracy.

### 2.4. Verification of the Numerical Methods

**Figure 2:**
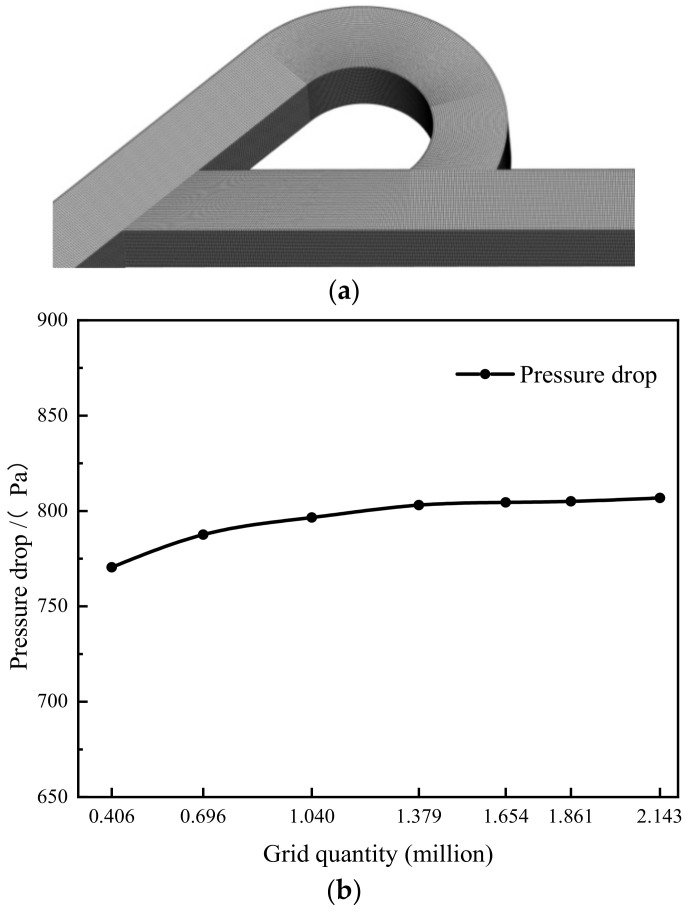
Computational mesh and grid-independence study. (**a**) Mesh for the single stage Tesla-type channel. (**b**) Pressure reduction between inlet and outlet under different grid densities.

As measured data of hydrogen through the Tesla-type channel is scarce, the experiment performed by Liu et al. [39] for water flowing through Tesla-type channel is used for model validation. Figure 2a shows a structured hexahedral mesh generated for the single stage Tesla-type channel used in the experiment. The mesh independence was verified using meshes with different numbers of cells. Figure 2b shows the predicted pressure reduction with increasing cell numbers. It was found that the pressure reduction did not change much when the number of cells increased to 1.379 million; therefore, the grid with 1.379 million cells was used for the validation simulation. To ensure the accuracy of simulation results, grid-independent verification has been carried out for all subsequent simulations.

**Figure 3:**
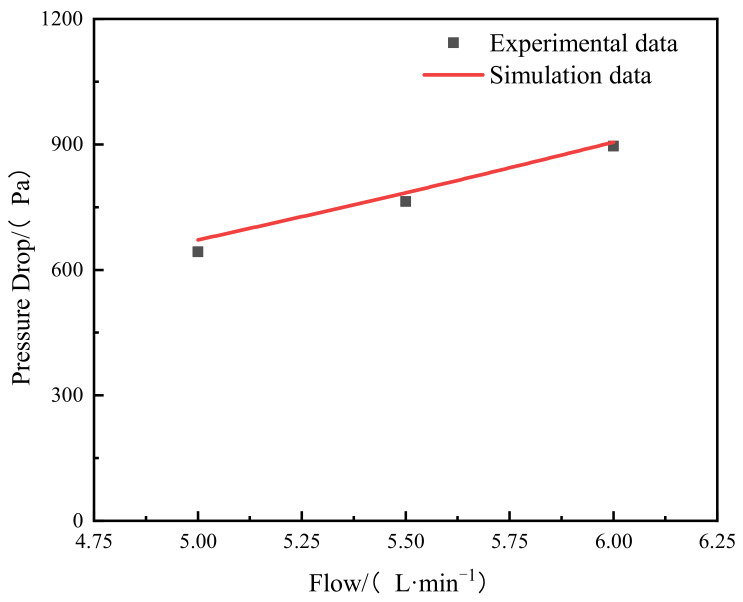
Pressure reduction: predicted vs. measured.

The experimental and simulated pressure reduction for different inlet flow rates are shown in Figure 3. It displaces a great consistency between prediction and measurement. The CFD model somewhat over-predicted the pressure reduction. The largest relative error between the results from simulation and observation is 4.48%, indicating that the CFD model can produce satisfactory prediction of fluid flow in a Tesla-type channel.

### 2.5. Computational Domain and Boundary Conditions

**Figure 4:**
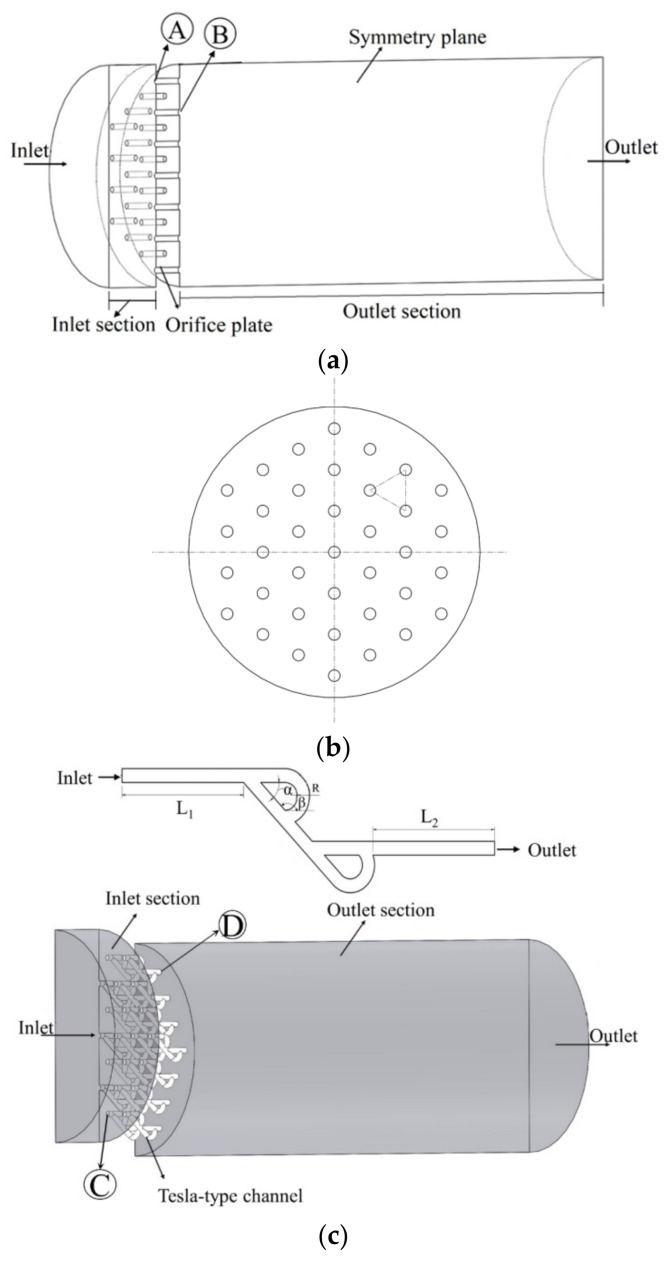

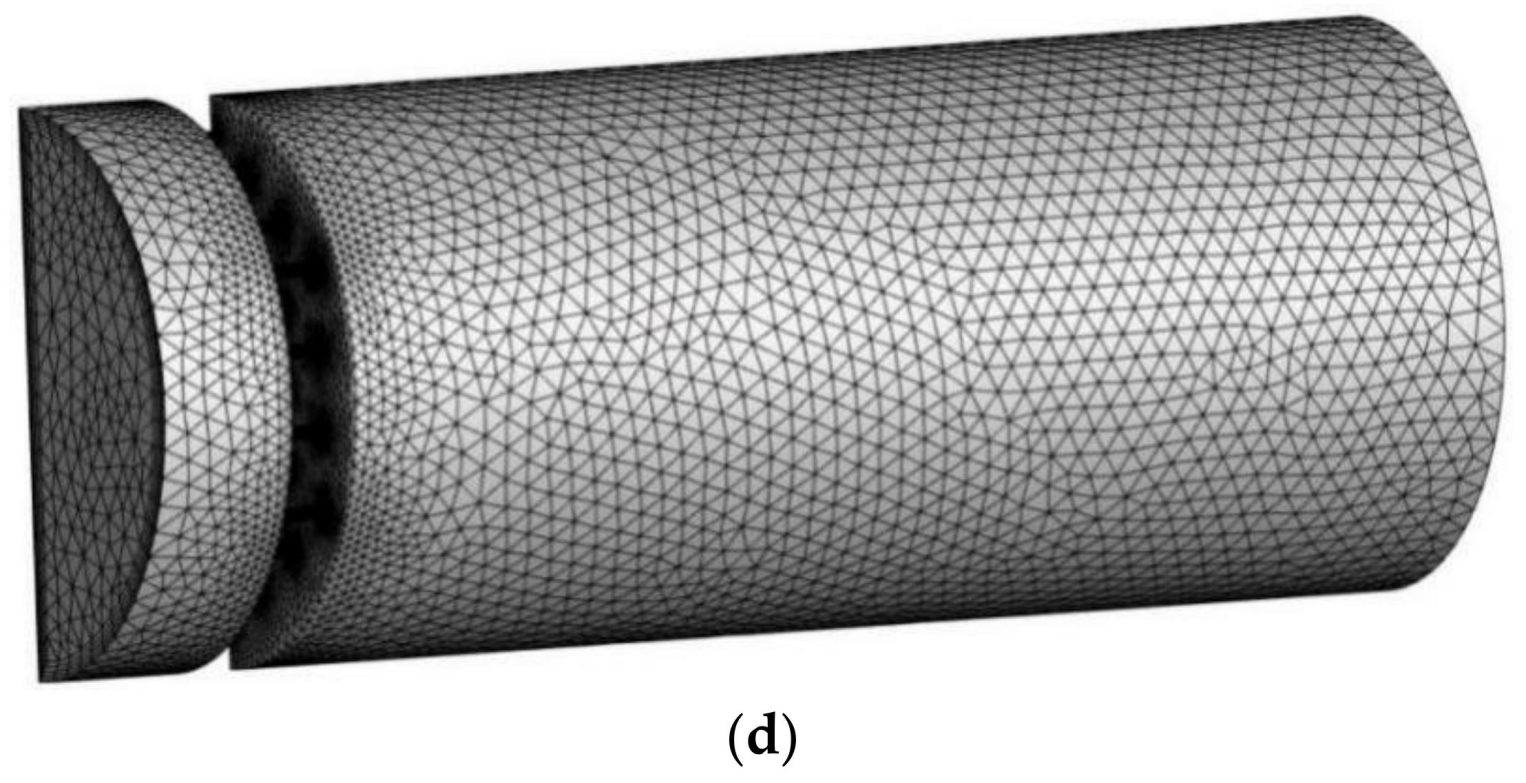
Computational domain and meshing. (**a**) Schematic of the fluid domain of conventional orifice plate structure. (**b**) The distribution of holes on the plate. (**c**) Schematic of fluid domain of the Tesla-type orifice structure. (**d**) Mesh division of Tesla-type orifice structure.

The benchmark structure of the traditional orifice plate valve was introduced and analyzed by Chen et al. [16,17]. For a better pressure reduction effect, Tesla-type channels are integrated into a conventional orifice plate structure to form a novel hydrogen depressurization structure. Figure 4a shows the structure of the traditional orifice plate valve. The central flow domain is a 200 mm diameter circular channel with a 50 mm long inlet section, a 450 mm long outlet section, and a 25 mm thick orifice plate. There are 37 holes on the plate which are staggered in equilateral triangles (see Figure 4b). In Figure 4a, Point A is where the orifice plate connected to the inlet section, while Point B is where the orifice plate connected to the outlet section. A Tesla-type channel usually has a good effect on pressure reduction. The pressure reduction performance can be further optimized when Tesla-type channels are integrated into conventional orifice plate structures. Figure 4c shows a modified structure which replaces the straight orifice flow channel with a Tesla-type channel in a traditional orifice plate. This Tesla-type orifice structure uses a circular channel with a diameter of 5 mm, so as to better couple with the main flow channel. Other structural parameters of the Tesla-type channel are: inlet length: *L*_1_ = 5 mm; outlet length: *L*_2_ = 5 mm; side straight channel length: *L* = 10 mm; the angle between side channel and main channel: *α* = 45°; the angle between bending channel and main channel: *β* = 130°; and the radius of the curve in the circular section: *R* = 2.5 mm. In Figure 4c, Point C is where the Tesla-type channel connected to the inlet section, and Point D is where the Tesla-type channel connected to the outlet section. Due to the symmetrical geometry, the computational domain uses half of the Tesla-type orifice structure.

The boundary conditions for conventional orifice plate structure and Tesla-type orifice structure are similar, which are defined as (see Figure 4): (a) inlet: mass flow inlet (flow rate *Q_m_*) with a constant temperature (300 K); (b) outlet: pressure outlet with a 0.2 MPa constant pressure; (c) symmetry plane: symmetrical impermeable boundary conditions with zero gradients of all variables; and (d) adiabatic wall boundary conditions specified to other boundary surfaces.

As the orifice plate has a relatively complex structure, a non-structural tetrahedral mesh was generated for the computational domain, as shown in Figure 4d. Mesh refinement was applied around the Tesla-type channel. Additionally, the energy residuals are 10 to the minus 6, and everything else is 10 to the minus 3. Key information of the model implementation is shown in Table 1.

## 3. Results and Discussion

### 3.1. Distribution of Pressure and Density

**Figure 5:**
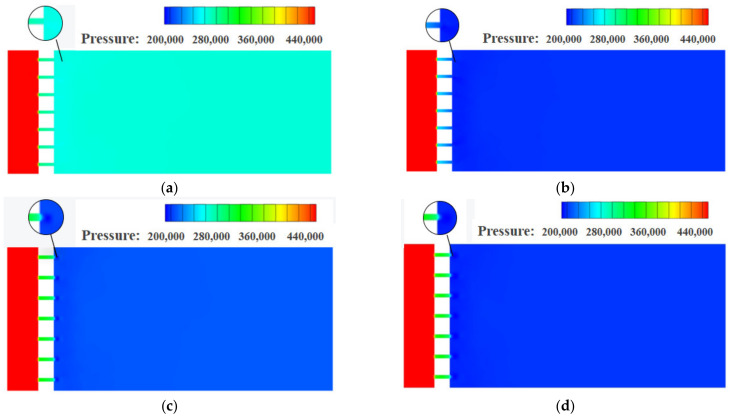
Pressure distribution on the symmetry plane for conventional orifice plate under different mass flow rates (pressure in Pascal). (**a**) *Q_m_* = 0.02 kg s^−1^. (**b**) *Q_m_* = 0.1 kg s^−1^. (**c**) *Q_m_* = 0.5 kg s^−1^. (**d**) *Q_m_* = 1 kg s^−1^.

**Figure 6:**
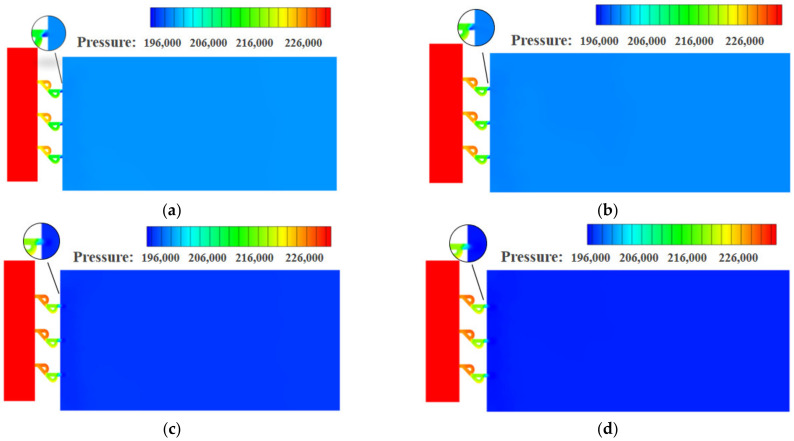
Pressure distribution on the symmetry plane for the Tesla-type orifice structure under different mass flow rates (pressure in Pascal). (**a**) *Q_m_* = 0.02 kg s^−1^. (**b**) *Q_m_* = 0.1 kg s^−1^. (**c**) *Q_m_* = 0.5 kg s^−1^. (**d**) *Q_m_* = 1 kg s^−1^.

The pressure distributions for traditional orifice plate structure and Tesla-type orifice structure on the symmetry plane under different inlet mass flow rates are shown in Figure 5 and Figure 6, respectively. It is found that the pressure reduction varies with inlet mass flow rate *Q_m_* for both structures. As shown in Figure 5a for the conventional orifice plate structure at *Q_m_* = 0.02 kg s^−1^, the maximum pressure gradient mainly occurs at Point A (refer to Figure 4a). At *Q_m_* = 0.1 kg s^−1^ (Figure 5b), the pressure begins to change dramatically at the location of the plate orifice. In general, there is a continuous pressure distribution between the orifice plate and outlet section when *Q_m_* is less than 0.1 kg s^−1^. When *Q_m_* increases to 0.5 kg s^−1^ or 1 kg s^−1^ (Figure 5c,d), step change in pressure gradient occurs at the connection between the orifice plate and outlet section. When *Q_m_* = 0.5 kg s^−1^ (Figure 5c), a small annular region with sudden pressure reduction is observed at Point B (refer to Figure 4a). The conventional orifice plate structure reduces the size of flow channel to throttle the hydrogen to achieve the pressure reduction. As shown in Figure 6, the Tesla-type orifice structure can achieve higher pressure reduction due to the increased resistance in the structure. Figure 6 shows that the pressure reductions are not only observed at connections between the Tesla channel and the inlet and outlet sections (Points C and D in Figure 4c), but also great pressure reduction can be seen between two stages of the Tesla channel. It is worth noting that the low-pressure zone at Point D shown in Figure 6a,b disappears with the rise of mass flow rate.

**Figure 7:**
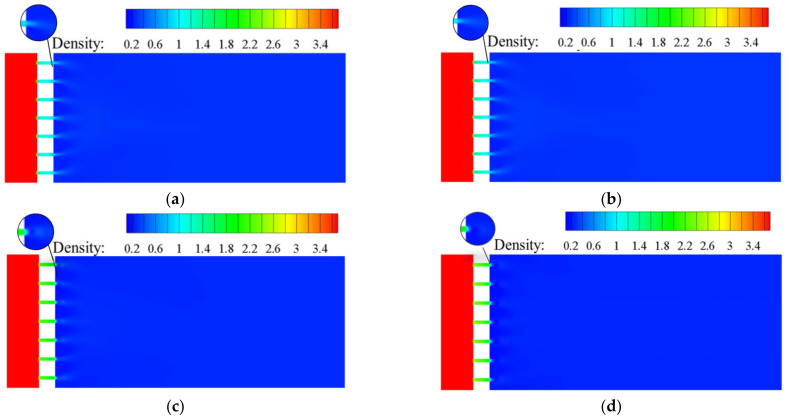
Density distribution on the symmetry plane for conventional orifice plate structure under different mass flow rates (density in kg m^−3^). (**a**) *Q_m_* = 0.02 kg s^−1^. (**b**) *Q_m_* = 0.1 kg s^−1^. (**c**) *Q_m_* = 0.5 kg s^−1^. (**d**) *Q_m_* = 1 kg s^−1^.

**Figure 8:**
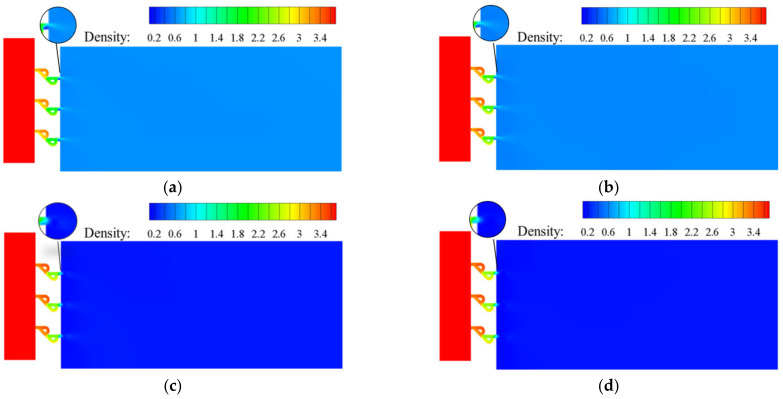
Density distribution on the symmetry plane for Tesla-type orifice structure under different mass flow rates (density in kg m^−3^). (**a**) *Q_m_* = 0.02 kg s^−1^. (**b**) *Q_m_* = 0.1 kg s^−1^. (**c**) *Q_m_* = 0.5 kg s^−1^. (**d**) *Q_m_* = 1 kg s^−1^.

Figure 7 and Figure 8 are the density distributions for the traditional orifice plate structure, as well as the Tesla-type orifice structure. The reduction in hydrogen pressure leads to the reduction in density. When the flow rate increases, the pressure and density gradients in the flow field increase. It is seen that the pressure greatly influences hydrogen density. This highlights the necessity of using real gas EoS in the simulation. In the Tesla-type orifice structure, the pressure varies in the channel due to the impact of the bending section. This is also reflected in the variation in the density.

### 3.2. Analysis of Mach Number and Turbulence Intensity

**Figure 9:**
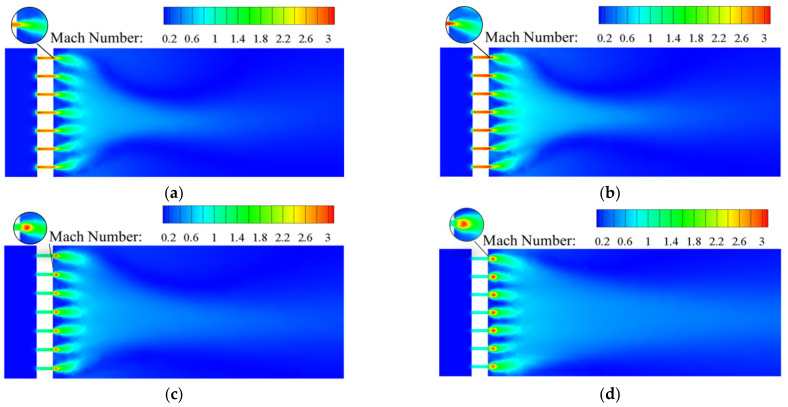
Mach number distribution on the symmetry plane for the conventional orifice plate structure under different mass flow rates. (**a**) *Q_m_* = 0.02 kg s^−1^. (**b**) *Q_m_* = 0.1 kg s^−1^. (**c**) *Q_m_* = 0.5 kg s^−1^. (**d**) *Q_m_* = 1 kg s^−1^.

Figure 9 shows the Mach number distribution in the conventional orifice plate structure under different inlet mass flow rate conditions. Similar Mach number distributions were observed when the inlet mass flow rates were *Q_m_* = 0.02 and 0.1 kg s^−1^. Under both mass flow rates, the Mach number is less than 1 throughout the flow domain. When hydrogen passes Point A, after adiabatic expansion, the pressure energy is converted into kinetic energy. This is reflected in the sudden decrease in the pressure and sharp rise in the velocity/Mach number. After entering the outlet section, the jet boundary is restricted by decreasing kinetic energy and velocity. The Mach number is distributed in such a way that the area near the wall is small and the area in the middle flow domain is large. As the mass flow rate rises to 1 kg s^−1^, the downstream jet flow affected area increases as well. When *Q_m_* = 0.02 kg s^−1^, Mach numbers in the traditional orifice plate structure and Tesla-type orifice structure are less than 0.3 while hydrogen behaves as a subsonic flow. When *Q_m_* rises to 0.5 or 1 kg s^−1^, the traditional orifice plate structure and the Tesla-type orifice structure have a supersonic flow at Point B and Point D. The hydrogen flows from the inlet section into the channel with abruptly decreasing area and then flows into the outlet section with much larger area. The flow is similar to that in a Laval nozzle [40].

**Figure 10:**
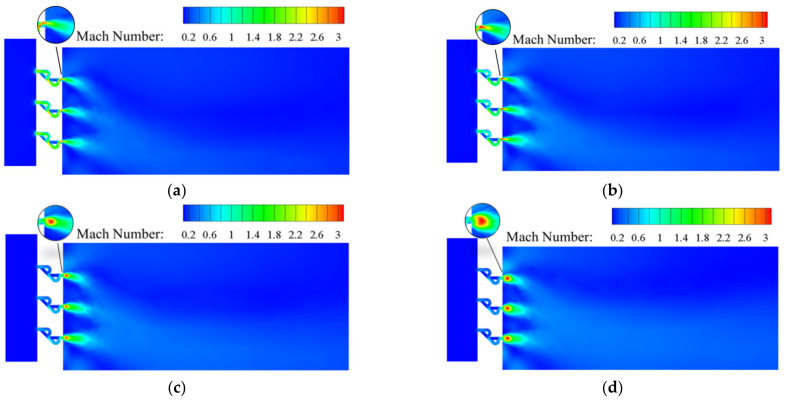
Mach number distribution on the symmetry plane for Tesla-type orifice structure under different mass flow rates. (**a**) *Q_m_* = 0.02 kg s^−1^. (**b**) *Q_m_* = 0.1 kg s^−1^. (**c**) *Q_m_* = 0.5 kg s^−1^. (**d**) *Q_m_* = 1 kg s^−1^.

As shown in Figure 9 and Figure 10, hydrogen is accelerated to the speed of sound in both the conventional orifice plate and the Tesla-type channels. Eventually, supersonic speed is achieved at the outlet section with an expanded cross-section. For both structures, it can be found that when *Q_m_* = 0.5 and 1 kg s^−1^, there is an area at Point B and Point D with low pressure and large Mach number. It is evident that expansion waves are generated here. In the Tesla-type orifice structure, the larger Mach number in the bending channel indicates that the velocity in the bending channel is higher than that in the straight channel, as the hydrogen flows more easily in the bending channel. When the hydrogen with high velocity flows out from the bending channel, it will impede the hydrogen in the straight channel, reducing the flow rate of hydrogen in the straight track and lowering the Mach number. The Mach number distribution in the outlet section of the Tesla-type orifice structure is clearly different from that of the conventional orifice plate structure. The Mach number near the lower part is larger than that in the upper part. The comparison between Figure 9 and Figure 10 demonstrates the area of the Tesla-type orifice structure with a Mach number greater than 1 is smaller than that of the traditional orifice plate structure. This indicates that the Tesla-type orifice structure reduces the area of fluid with high velocity, which can also help achieve better pressure reduction.

**Figure 11:**
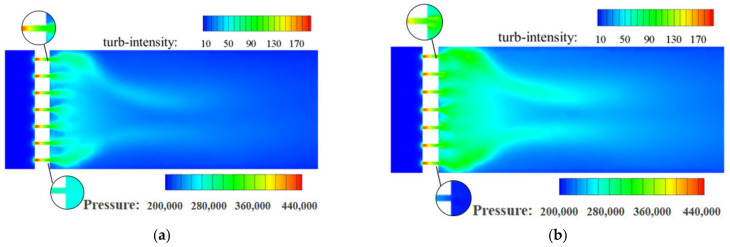

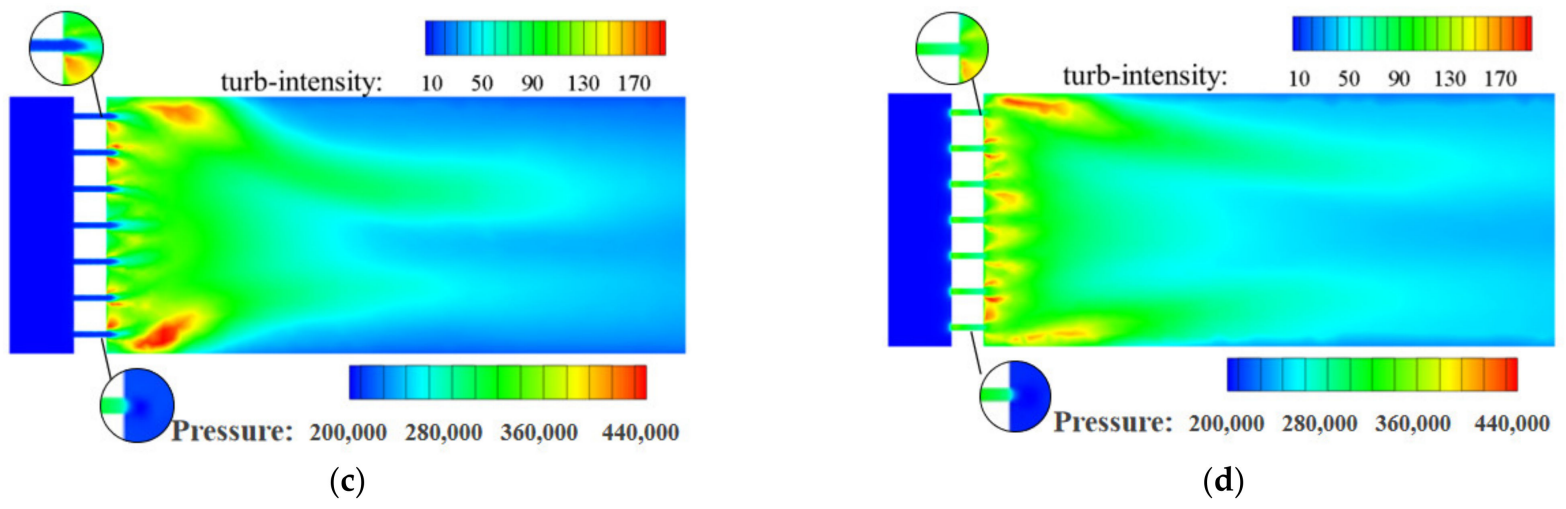
Turbulence intensity distribution on the symmetry plane for the conventional orifice plate structure under different mass flow rates (%). (**a**) *Q_m_* = 0.02 kg s^−1^. (**b**) *Q_m_* = 0.1 kg s^−1^. (**c**) *Q_m_* = 0.5 kg s^−1^. (**d**) *Q_m_* = 1 kg s^−1^.

**Figure 12:**
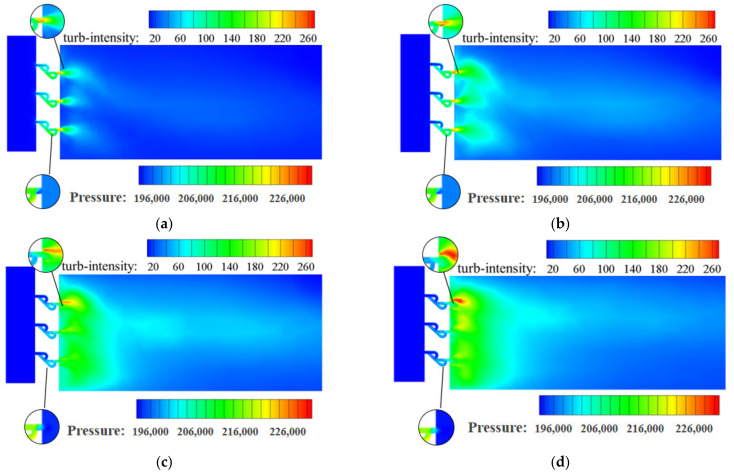
Turbulence intensity distribution on the symmetry plane for the Tesla-type orifice structure under different mass flow rates (%). (**a**) *Q_m_* = 0.02 kg s^−1^. (**b**) *Q_m_* = 0.1 kg s^−1^. (**c**) *Q_m_* = 0.5 kg s^−1^. (**d**) *Q_m_* = 1 kg s^−1^.

Figure 11 and Figure 12 show the turbulence intensity on the symmetry plane of the conventional orifice plate structure and the Tesla-type orifice structure, respectively. We can see that the maximum turbulence intensity increases with the inlet mass flow rates. As *Q_m_* reaches 0.02 and 0.1 kg s^−1^, the maximum turbulence intensity of the conventional orifice plate structure appears at Point A. The turbulence intensity at Point B is much higher than that at the end of outlet section. As *Q_m_* = 0.02 kg s^−1^, the jet of each plate orifice at the exit has less influence on each other. The turbulence intensity between the two plates is smaller and the velocity of the hydrogen jet is lower. Additionally, the velocity gradient in this area is low because the diversion effect between the two jets of the plate orifice is small. When *Q_m_* = 0.1 kg s^−1^, the area of low turbulence intensity zone between the plate orifice exits decreases as the plate orifice exit velocity becomes larger. As *Q_m_* reaches 0.5 and 1 kg s^−1^, the maximum turbulence intensity of the conventional orifice plate structure appears at Point B and near the wall.

As shown in Figure 12, the maximum turbulence intensity in the Tesla-type orifice structure appears at Point D. The maximum turbulence intensity is observed at the uppermost plate orifice exit when a supersonic flow occurs there. The turbulence intensity is more significant in the exit section of the Tesla-type orifice structure near the upper wall. It is found that changes in hydrogen flow rate, as well as changes in the structure, affect turbulence intensity. Comparison of Figure 11 and Figure 12 shows that the Tesla-type orifice structure is easier to enable the formation of vortices due to higher turbulent intensity induced; this is mainly because the Tesla-type orifice structure makes it easier for the fluid to enter the bending channel. The interaction between the fluid in straight and the bending channel results in the increase in turbulent intensity and also leads to the abrupt pressure drop.

## 4. Optimization of Tesla-Type Orifice Structure

### 4.1. Optimization Methods

**Figure 13:**
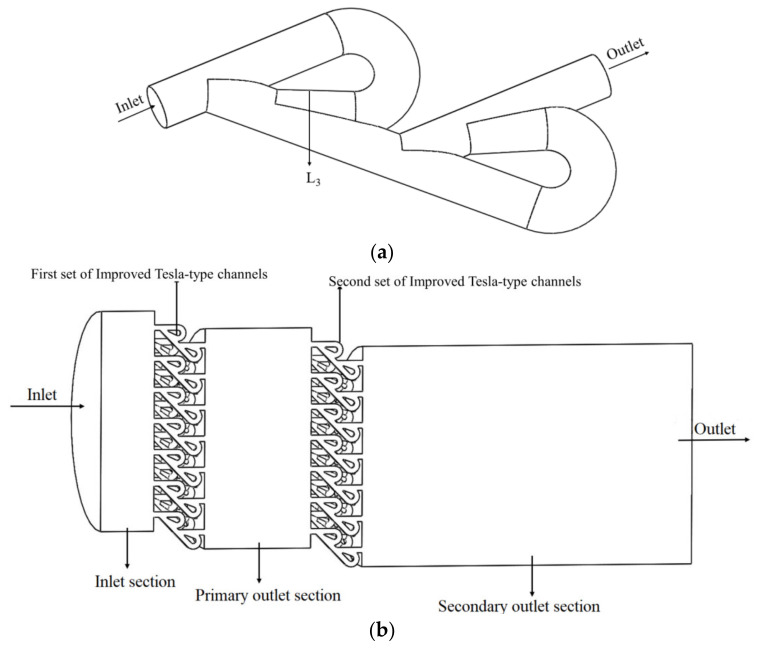
Improved Tesla-type channel and two-stage Tesla-type orifice structure. (**a**) Improved Tesla-type channel. (**b**) Two-stage Tesla-type orifice structure.

The above study shows that a higher pressure-reduction performance can be achieved by replacing the conventional orifice plate structure with a simple orifice plate integrated with a Tesla-type orifice structure flow channel. In order to achieve a better performance on pressure reduction, the structure of a Tesla-type orifice can be further optimized by improving the Tesla-type channel. Figure 13a shows the improved Tesla-type orifice structure flow path. The pressure reduction performance of the Tesla-type orifice structure is investigated. The above study illustrates the main reason that a Tesla valve can reduce pressure is that its bending channel has an impeding effect on the flow in the straight channel. To enhance the flow impeding effect, a novel construction was introduced at the junction of the bending and straight channels. In the new structure, the flow in the bending channel was brought to interact with the flow in the straight channel earlier (see Figure 13a). The angle *β* between the main channel and the bent channel was increased. These modifications increase the impeding effect of the return flow in bending channel. In the new structure, *L*_3_ = 8.6 mm (Figure 13a).

In addition to the improvement in the Tesla-type channel, another set of parallel Tesla-type channels was introduced to the Tesla-type orifice structure, which forms a two-stage Tesla-type orifice structure, as shown in Figure 13b. The lengths of the inlet section, the primary outlet section, and the secondary outlet section are 50, 100, and 200 mm, respectively. The main flow channel is also a circular channel with a diameter of 200 mm. Additionally, half of the proposed structure is used as the computational domain (Figure 13b).

To evaluate the pressure reduction performance of the improved Tesla-type orifice structure, the same mass flow rates of 0.01, 0.1, 0.5, and 1 kg s^−1^ and outlet pressure of 0.2 MPa are used for the CFD simulations.

### 4.2. Flow Field Analysis of the Two-Stage Tesla-Type Orifice Structure

**Figure 14:**
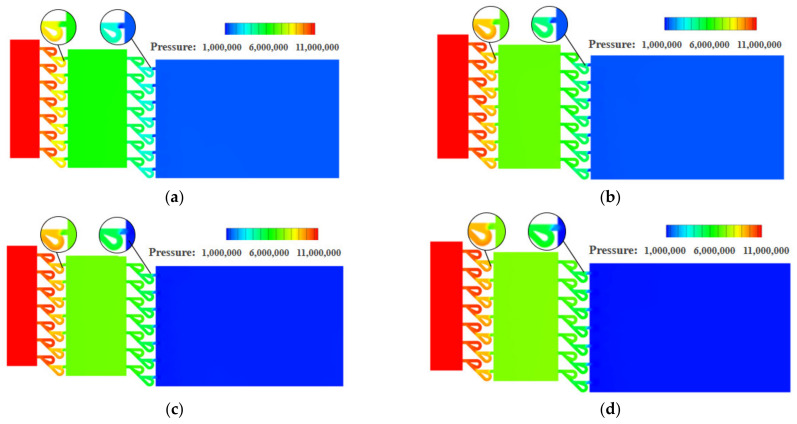
Pressure distribution on the symmetry plane for the two-stage Tesla-type orifice structure under different mass flow rates (Pressure in Pascal). (**a**) *Q_m_* = 0.02 kg s^−1^. (**b**) *Q_m_* = 0.1 kg s^−1^. (**c**) *Q_m_* = 0.5 kg s^−1^. (**d**) *Q_m_* = 1 kg s^−1^.

Figure 14 shows the pressure distribution on the symmetry plane of the two-stage Tesla-type orifice structure under different inlet mass flow rates. It is found that the pressure in the orifice structure reduces several times. In addition to the pressure reduction when entering the Tesla-type flow channel orifice in the inlet section, a significant pressure reduction can be seen each time when hydrogen flows through the junction of the bending and straight channels. In each outlet section of the two-stage Tesla-type orifice structure, the pressure of hydrogen is evenly distributed with minimal variation. As the mass flow rate increases, the pressure reduction in the orifice structure increases continuously. Compared to Figure 6, it is obvious that the improved Tesla-type orifice structure achieved better pressure reduction performance. Additionally, the pressure in the improved Tesla-type orifice structure was reduced much more smoothly.

**Figure 15:**
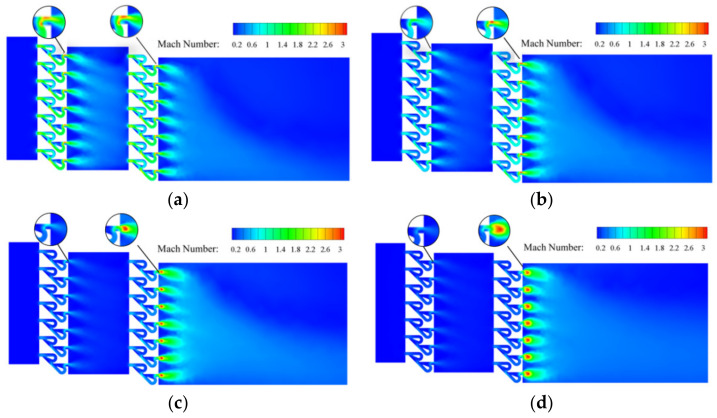
Mach number distribution on the symmetry plane for the two-stage Tesla-type orifice structure under different mass flow rates. (**a**) *Q_m_* = 0.02 kg s^−1^. (**b**) *Q_m_* = 0.1 kg s^−1^. (**c**) *Q_m_* = 0.5 kg s^−1^. (**d**) *Q_m_* = 1 kg s^−1^.

The Mach number distributions are shown in Figure 15. As the inlet mass flow rate increases, the maximum Mach number in the two-stage Tesla-type orifice structure gradually increases. When *Q_m_* = 0.02 kg s^−1^, the largest Mach number appears behind the bending channel of the second stage. This is caused by the sharp decrease in pressure and the rise in velocity at this location because of the hydrogen in the bending channel joining the hydrogen from the straight channel. There is limited difference in Mach numbers between the two stages of Tesla-type channels. When *Q_m_* = 0.1 kg s^−1^, the maximum Mach number appears at the rear position of the bending channel of the second stage of the new orifice channels. This indicates that the velocity is higher at the second stage of Tesla-type orifice structure, implying more pressure reduction. When *Q_m_* rises to 0.5 and 1 kg s^−1^, a supersonic flow occurs in the two-stage Tesla-type orifice structures. The hydrogen gas flows out of the second stage Tesla-type channels with a sudden increase in cross-section area. An expansion wave is generated, causing a sudden increase in flow velocity, with maximum Mach numbers observed near the channel exits.

**Figure 16:**
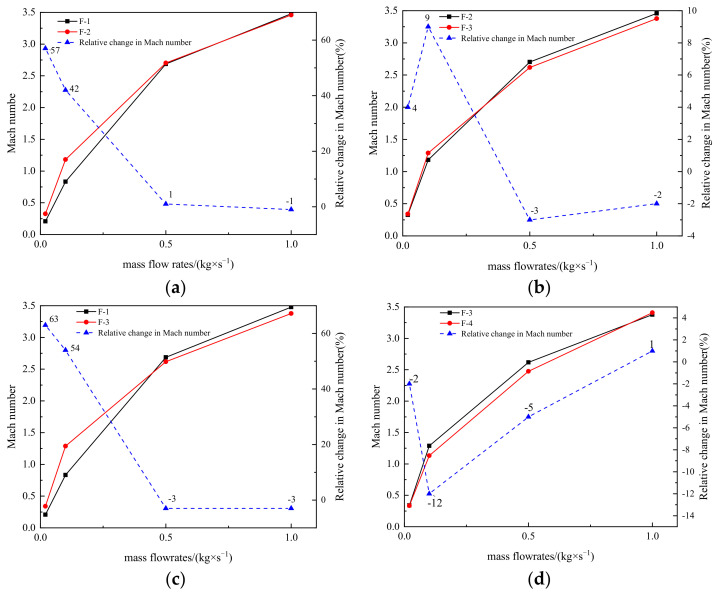
Relative change in Mach number of four structures under different mass flow rates. (**a**) Relative change in Mach number between F-1 and F-2. (**b**) Relative change in Mach number between F-2 and F-3. (**c**) Relative change in Mach number between F-1 and F-3. (**d**) Relative change in Mach number between F-3 and F-4.

Figure 16 shows the relative magnitude of maximum Mach number for four types of valves under different mass flow rates. In Figure 16, F-1 represents the conventional orifice plate structure, F-2 the original Tesla-type orifice structure, F-3 the one-stage improved Tesla-type orifice structure, and F-4 the two-stage optimized Tesla-type orifice structure. Figure 16a shows that, when the traditional orifice plate structure is replaced by the Tesla-type orifice structure, up to 0.5 kg s^−1^ mass flow rate, there is a relatively large growth in Mach number; however, a further increase in the mass flow rate causes very limited improvement in the Mach number. When *Q_m_* = 1 kg s^−1^, the Mach number of Tesla-type orifice structure becomes smaller than that of the conventional orifice plate structure. A similar trend is demonstrated in Figure 16c, but the growth in mass flow rate will result in a greater reduction in Mach number for the one-stage improved Tesla-type orifice structure. Figure 16b demonstrates that the one-stage improved Tesla-type orifice structure initially shows a larger Mach number than the original Tesla-type orifice structure; however, the growth in the mass flow rate will lead to a Mach number smaller than that of the original Tesla-type orifice structure. Figure 16d shows that, when the mass flow rate is less than 0.1 kg s^−1^, the two-stage Tesla-type orifice structure has a slightly larger Mach number than the one-stage one. However, the increase in the mass flow rate will soon cause a smaller Mach number for the two-stage Tesla-type orifice structure. Overall, for most of the mass flow rates, the two-stage Tesla-type orifice structure shows a smaller maximum Mach number. This proves that the two-stage Tesla-type orifice structure is an effective way to obtain the same pressure reduction with lower Mach number.

**Figure 17:**
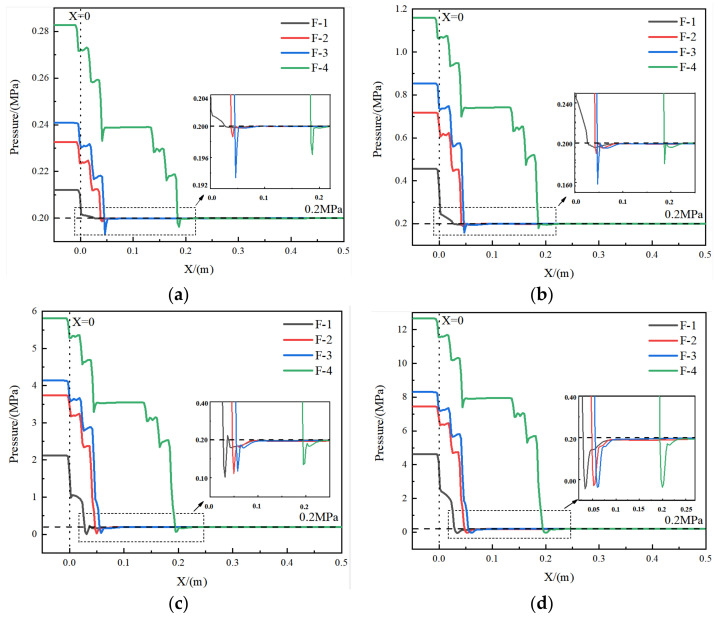
Pressure distribution on the centerline of the symmetry plane for the four structures under different mass flow rates. (**a**) *Q_m_* = 0.02 kg s^−1^. (**b**) *Q_m_* = 0.1 kg s^−1^. (**c**) *Q_m_* = 0.5 kg s^−1^. (**d**) *Q_m_* = 1 kg s^−1^.

Figure 17 demonstrates the pressure on the centerline of the symmetry plane of the four structures. The change in hydrogen pressure due to the difference in the structure is well reflected. In Figure 17, X = 0 corresponds to where the inlet section is connected to the plate orifice and positive X corresponds to the flow direction of hydrogen. It is shown in Figure 17 that, for all four types of pressure reduction valves, higher inlet mass flow rate leads to higher pressure reduction. Among these valves, the conventional orifice plate structure has the worst pressure reduction performance. Comparison between the original and the optimized Tesla-type orifice structures shows that the first stage pressure reduction is formed when the hydrogen enters the plate orifice, and the subsequent two stages of pressure reduction is due to the flow channel characteristics of Tesla valve structure. The investigation reveals that the optimized Tesla-type orifice structure does improve the effect of impeding flow and obtained better pressure reduction performance.

It can also be seen from the figure that, for the Tesla-type valves, as *Q_m_* reaches 0.02 and 0.1 kg s^−1^, the hydrogen pressure experiences a recovery before the hydrogen enters the outlet section. This is different from the pressure recovery for *Q_m_* = 0.5 and *Q_m_* = 1 kg s^−1^. When *Q_m_* = 0.5 and *Q_m_* = 1 kg s^−1^, low-pressure sectors are formed due to the expansion when hydrogen enters the outlet section, and the pressure recovery curve is smoother. In contrast, when *Q_m_* = 0.02 and 0.1 kg s^−1^, the pressure recovery is due to the vortex formed at the intersection of the bending and straight channels, and the pressure recovery curve is sharper than that for higher inlet mass flow rates.

**Figure 18:**
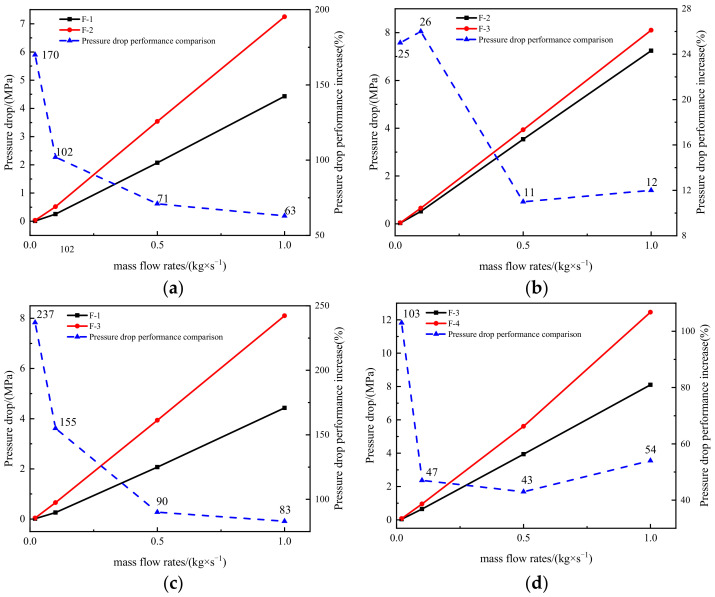
Comparison of pressure reduction performance of four structures. (**a**) F-1 and F-2 pressure reduction. (**b**) F-2 and F-3 pressure reduction. (**c**) F-1 and F-3 pressure reduction. (**d**) F-3 and F-4 pressure reduction.

Figure 18 shows the comparison of the pressure reduction performance of the four structures at different inlet mass flow rates. The pressure reduction is enhanced when the straight channel in the traditional orifice plate structure is replaced by the Tesla valve flow channel (Figure 18a). When *Q_m_* reaches 0.02 kg s^−1^, there is an up to 170% increase in the magnitude of pressure reduced. As the mass flow rate increases, the relative increase in the magnitude of pressure reduction across the Tesla-type orifice structure decreases. At *Q_m_* = 0.5 kg s^−1^, the downward trend slows down after the appearance of the supersonic flow. Similarly, the improved Tesla-type orifice structure achieved better pressure reduction performance than the conventional orifice plate structure. However, at *Q_m_* = 0.02 kg s^−1^, it achieved a 237% increase in the magnitude of pressure reduced compared to the conventional orifice plate structure.

In Figure 18b, the pressure reduction performance between the original and improved Tesla-type orifice structures is compared. It indicates that the one-stage improved Tesla-type orifice structure achieved further pressure reduction. For *Q_m_* < 0.1 kg s^−1^, the growth rate of the relative pressure reduction in the one-stage improved Tesla-type orifice structure becomes more significant than that of the original Tesla-type orifice structure. When supersonic flow presents in the orifice structure, the relative increase in the magnitude of pressure reduction in the one-stage improved Tesla-type orifice structure shrinks. As shown in Figure 18d, the two-stage improved Tesla-type orifice structure shows obvious improvement in the pressure reduction performance compared to the one-stage one. However, the relative increase in the pressure reduction magnitude reduces with the increase in mass flow rate. When supersonic flow forms in the valve, the relative increase in the pressure reduction magnitude reduces to its minimum value. Under this situation, increase in the mass flow rate will result in the increase in pressure reduction magnitude. Overall, it is found that the improved Tesla valve is able to achieve better pressure reduction performance than the original Tesla valve. Under a low inlet mass flow rate, a second stage of Tesla valve can be introduced to further improve the pressure reduction performance.

## 5. Conclusions

In this work, a novel Tesla-type orifice structure used for high-pressure hydrogen depressurization in HFCVs is proposed. The cylindrical channel in a traditional orifice plate structure is replaced by a Tesla valve flow channel. It is found that the pressure reduction performance could be improved significantly without a significant increase in size. The flow impeding effect of the Tesla-type orifice structure is primarily responsible for the pressure reduction improvement. To enhance the flow impeding effect, modifications are introduced to the Tesla-type channel and the pressure reduction performance has been further improved. It can be concluded that:

(1) In contrast to the conventional orifice structure, the Tesla-type orifice structure has a better performance on pressure reduction. Modifications introduced to the Tesla channel can further improve the pressure reduction performance. Under an inlet mass flow rate of 0.02 kg s^−1^, the pressure reduction can be increased by 237% compared to the conventional orifice structure;
(2) To further improve the pressure reduction performance, a second set of Tesla-type channels can be introduced to form a two-stage Tesla-type orifice structure. Additionally, the angle β between the bent channel and the main channel increased by more than 130° and L_3_ reduced to 8.6 mm in the two-stage Tesla-type orifice structure;
(3) Under the same mass flow rate, the maximum Mach number in the Tesla-type orifice structure is greater than that in the conventional orifice plate structure before the occurrence of supersonic flow. A lower Mach number can alleviate the start-up noise of fluid flow and save energy. When the supersonic flow is formed, the Tesla-type orifice structure shows a similar or smaller maximum Mach number. The two-stage Tesla-type orifice structure can effectively reduce the maximum Mach number with the same pressure reduction;
(4) Due to the asymmetry of the Tesla-type orifice structure, hydrogen flows towards the lower wall when entering the outlet section, producing a wall-fitting effect on the lower wall surface. The vortex can lead to mechanical energy consumption because it generally aggravates the turbulence of the hydrogen flow. A large vortex is formed in the upper area of the outlet chamber with a low turbulence intensity. In contrast to the traditional orifice plate structure, the Tesla-type orifice structure shows less vortices in the high turbulence intensity region, reducing energy consumption.

## Figures and Tables

**Table 1:** Key information of the model implementation.

Computational Time	Number of Iterations	Convergence Criteria
40–80 h/case	20	1 × 10^−6^/1 × 10^−3^

## Nomenclature

*C*_2_ *C*_1ε_	Constants
*E*	Total energy per unit control body
***g***	Gravitational acceleration
*G_k_*	Generation of turbulence kinetic energy due to the mean velocity gradients
*G_b_*	Generation of turbulence kinetic energy due to buoyancy
*k_eff_*	Effective thermal conductivity
*L*	Side straight channel length
*L*_1_	Inlet length
*L*_2_	Outlet length
***p***	Pressure vector
*P_c_*	Critical pressure
*Q_m_*	Flow rate
*R*	Universal gas constant
*t*	Time
*T_c_*	Critical temperature
***v***	Velocity vector
*v*	Molar volume
*Y_M_*	Contribution of the fluctuating dilatation incompressible turbulence to the overall dissipation rate
Greek symbols	
*α*	Angle between side channel and main channel
*β*	Angle between bending channel and main channel
*ρ*	Gas density
*τ*	Viscous stress tensor
*σ_k_ σ_ε_*	Turbulent Prandtl numbers for *k* and *ε*
*ω*	Eccentricity factor of the gas
Abbreviations list	
EoS	Equation of State
PHP	Pulsating Heat Pipe
2D/3D	two-dimensional/three-dimensional
PR	Peng–Robinson
GERG-2008	Groupe Européen de Recherches Gazières
HMPRV	High-level Multistage PRV
CFD	Computational Fluid Dynamic
PRVs	Pressure Reducing Valves
HFCVs	Hydrogen Fuel Cell Vehicles
